# Non-collinear spin states in bottom-up fabricated atomic chains

**DOI:** 10.1038/s41467-018-05364-5

**Published:** 2018-07-20

**Authors:** Manuel Steinbrecher, Roman Rausch, Khai Ton That, Jan Hermenau, Alexander A. Khajetoorians, Michael Potthoff, Roland Wiesendanger, Jens Wiebe

**Affiliations:** 1 Department of Physics, Hamburg University, Jungiusstrasse 9A, 20355 Hamburg, Germany; 2 Institute for Molecules and Materials (IMM), Radboud University, P.O. Box 9010//078, 6500 GL Nijmegen, The Netherlands; 3 I. Institute for Theoretical Physics, Hamburg University, Jungiusstrasse 9, 20355 Hamburg, Germany

## Abstract

Non-collinear spin states with unique rotational sense, such as chiral spin-spirals, are recently heavily investigated because of advantages for future applications in spintronics and information technology and as potential hosts for Majorana Fermions when coupled to a superconductor. Tuning the properties of such spin states, e.g., the rotational period and sense, is a highly desirable yet difficult task. Here, we experimentally demonstrate the bottom-up assembly of a spin-spiral derived from a chain of iron atoms on a platinum substrate using the magnetic tip of a scanning tunneling microscope as a tool. We show that the spin-spiral is induced by the interplay of the Heisenberg and Dzyaloshinskii-Moriya components of the Ruderman-Kittel-Kasuya-Yosida interaction between the iron atoms. The relative strengths and signs of these two components can be adjusted by the interatomic iron distance, which enables tailoring of the rotational period and sense of the spin-spiral.

## Abstract (web summary)

Scanning tunnelling microscopes can be used to accurately position atoms and measure emergent behaviour arising from interatomic couplings. Here, the authors fabricate a model spin chain and show the formation of a tunable spiral state due to competing Heisenberg and Dzyaloshinskii-Moriya interactions

## Introduction

Ultra-thin layers and atomic chains of transition metal atoms on high-*Z* substrates show a variety of non-collinear spin states^1^ ranging from chiral domain walls^2^ over spin-spirals^3–8^ to skyrmions^9^. In most of the cases, these non-collinear spin states are stabilized by the so-called Dzyaloshinskii-Moriya (DM) interaction ${\mathbf{D}}_{ij}( {{\hat{\mathbf S}}_i \times {\hat{\mathbf S}}_j})$ between neighboring spins ${\hat{\mathbf S}}_i$ and ${\hat{\mathbf S}}_j$, which is relevant due to the broken inversion asymmetry at the interface of the magnetic material and the substrate. While the usual Heisenberg interaction $J_{ij}\,{\hat{\mathbf S}}_i \cdot {\hat{\mathbf S}}_j$ favors a parallel (ferromagnetic) or antiparallel (antiferromagnetic) alignment of neighboring spins, the DM interaction favors perpendicular orientations. The interplay of these two interactions can induce a spin-spiral state, with a rotational sense determined by the orientation of **D**_*ij*_, and with a period given by the ratio of *D*_*ij*_ and *J*_*ij*_, and by the magnetic anisotropy^10^.

For many of the proposed applications of such chiral spin-spiral states, e.g., in spintronics and information technology^1,2,5^ or as platforms for Majorana quasiparticles^11–14^, a tuning of these parameters is highly desirable, but challenging^15,16^. A very appealing approach towards this end is to use the tip of a scanning tunneling microscope to construct chains atom by atom with tailored magnetic anisotropy and interactions. While numerous bottom-up magnetic chains have been investigated^17–22^, these studies have focused solely on Heisenberg interactions inducing collinear order. However, the ubiquitous Ruderman-Kittel-Kasuya-Yosida (RKKY) interaction between magnetic atoms in contact to metallic substrates additionally contains a DM component leading to non-collinear order, which is typically strong for high-*Z* materials^23,24^. Indeed, it has been shown recently that the DM component of the RKKY interaction between Fe atoms on Pt(111) is of comparable strength as the Heisenberg part^25^. Most notably, due to the oscillatory nature of the RKKY coupling, the two components are adjustable in their relative strengths and signs by changing the distance between the Fe atoms^25^. The use of similar material combinations of dilute transition metal chains on high-*Z* substrates therefore promises the stabilization of adjustable spin-spiral states in bottom-up fabricated chains.

Here, we have experimentally realized this idea in the form of a spin-spiral state residing in a 16 atom long Fe chain that has been assembled atom-by-atom on the (111) surface of a Pt single crystal. By comparison of the experimental results to density-matrix renormalization group (DMRG) calculations, we demonstrate that the spin-spiral’s wavelength and rotational sense can be tuned via the interatomic distances of the Fe atoms in the chain.

## Results

### Construction of chains of various lengths

**Fig. 1:**
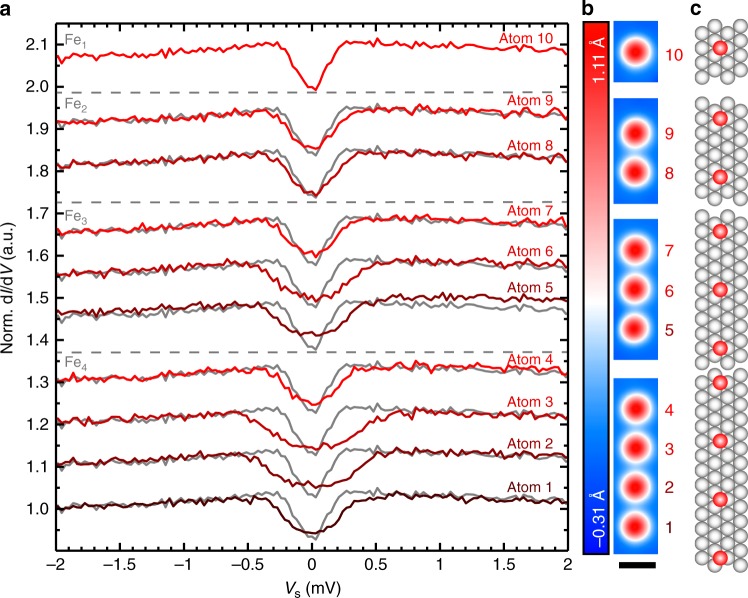
ISTS of bottom-up Fe_*N*_ chains of different numbers *N* of atoms. **a** ISTS spectra taken on top of an isolated Fe atom (Fe_1_, 10) and on the Fe atoms inside Fe_2_ (8, 9), Fe_3_ (5, 6, 7), and Fe_4_ (1, 2, 3, 4) chains with *d*_NN_ = 4*a*. For comparison, the spectrum of the isolated Fe_1_ is shown in gray behind all other spectra. All spectra were normalized by dividing by a substrate spectrum. (*V*_stab_ = −6 mV, *I*_stab_ = 3 nA, *V*_mod_ = 40 μV). **b**, **c** STM topographs and schematic ball models where the red and gray spheres represent the Fe and surface Pt atoms, respectively, of all chains investigated in (**a**) (*V*_s_ = −6 mV, *I*_stab_ = 0.5 nA). The black scale bar below (**b**) has a length of 1 nm

Using tip-induced atom manipulation we built Fe_*N*_ chains on a Pt(111) surface with different numbers *N* of Fe atoms and with different nearest neighbor (NN) distances *d*_NN_ (see 'Methods' for experimental details). Figure 1b and Supplementary Fig. 1 show an isolated Fe atom (Fe_1_) and assembled Fe_*N*_ chains with *N* = 2, 3, 4 and *d*_NN_ = 4*a* with the shortest distance of equivalent hollow adsorption sites of *a* = 2.78 Å. All chains investigated in this work were built from Fe atoms sitting on the hexagonal close-packed (hcp) adsorption site, as identified by the characteristic spin-excitation at 0.19 meV in inelastic scanning tunneling spectroscopy (ISTS) taken on Fe_1_ (Fig. 1a). As shown in ref. ^26^, they exhibit a relatively weak easy-plane magnetic anisotropy, which only slightly favors an orientation of the Fe spin in the surface plane. From an investigation of pairs of an Fe-hydrogen complex and an Fe atom with different distances^25^, we expect that the chosen *d*_NN_ results in an antiferromagnetic Heisenberg interaction of *J*_NN_ ≈ −50 μeV and a comparable DM coupling *D*_⊥,NN_ ≈ +30 μeV between NN Fe atoms in the chains (see Supplementary Note 1 and Supplementary Fig. 2 for the definition of the coordinates).

### Non-collinear spin states in 4*a* chains

ISTS spectra taken on the Fe atoms in the Fe_*N*_ chains (Fig. 1a) reveal considerable shifts of the spin-excitation to higher excitation energies and a broadening of the excitation step as compared to the isolated Fe_1_ shown in gray as a reference^25^. This effect is more pronounced for the atoms with two neighbors (atoms 2, 3, 6) and we therefore tentatively assign the shifts to the mutual magnetic interactions between the atoms, which can be used to determine the corresponding coupling parameters with an accuracy of 10 μeV by comparison to model calculations^25^. Note, that there is a negligible residual interaction between the chains leading to a slight asymmetry within each chain (see Supplementary Fig. 1) which results in the tiny nonequivalence of the two end atoms (Fig. 1a). Due to symmetry arguments, the DM vector has its largest component in the surface plane perpendicular to the chain axis $( {D_{||} = 0,D_z \,{\mathrm{small}}} )$^25^. We therefore anticipate a tendency towards the formation of a cycloidal spin-spiral state in the (||, *z*)-plane. Since the chain atoms are strongly coupled to the substrate conduction electrons, these states typically do not show remanence^5,19^ and therefore cannot be resolved by conventional spin-polarized (SP) scanning tunneling microscopy (STM) in the absence of a magnetic field. We will therefore use two different methods, namely (i) an external homogeneous magnetic field *B*_*z*_ along the surface normal (*z*) and (ii) RKKY coupling one end of the chain to a ferromagnetic island^27,28^, in order to stabilize the non-collinear spin states.

**Fig. 2:**
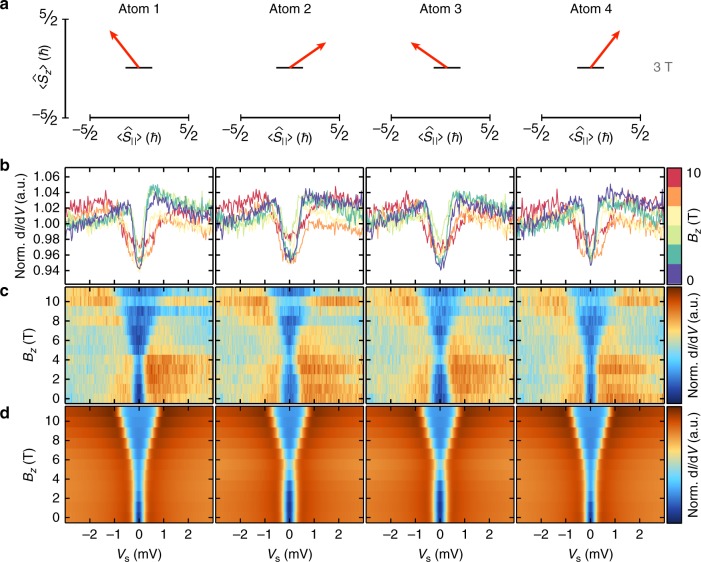
Non-collinear spin state in the Fe_4_ chain with *d*_NN_ = 4*a*. **a** Calculated spin-expectation values $( {\langle {\hat S_{||}} \rangle ,\langle {\hat S_ \bot } \rangle ,\langle {\hat S_z} \rangle } )$, see arrows ($\langle {\hat S_ \bot } \rangle = 0$ by symmetry), for each atom in the Fe_4_ chain at *B*_*z*_ = 3 T. The results are obtained by exact diagonalization (ED) of a spin model with parameters given in Table 1, *J*_NN_ = −25 μeV, and *D*_⊥,NN_ = +30 μeV. **b**, **c** ISTS spectra (**b**) and corresponding colorplots (**c**) measured on all four atoms in the Fe_4_ chain for different magnetic fields *B*_*z*_ up to *B*_*z*_ = 11 T. (*V*_stab_ = −6 mV, *I*_stab_ = 3 nA, *V*_mod_ = 40 μV). **d** Calculated ISTS from the perturbation theory model using the same parameters as in (**a**)

First, we applied *B*_*z*_ to the chains and performed ISTS. For the sake of simplicity, we will focus on the data of the Fe_4_ chain shown in Fig. 2b, c (ISTS and calculations of all other chains are shown in Supplementary Figs. 3–6 as described in Supplementary Note 2 showing the reproducible evolution of the spectra from *N* = 2 to 4). The spectra reveal a small shift of the spin-excitation to lower energies for field strengths up to *B*_*z*_ ≈ 4, and then a nearly linear increase in the spin-excitation energy proportional to *B*_*z*_, with slight differences between the spectra on the two inner and the two edge atoms of the chain. Compared to the *B*_*z*_-dependent ISTS of the Fe_1_, where the minimum of the excitation energy appears at *B*_*z*_ ≈ 3 T ^26^ (see Supplementary Fig. 4a), this minimum is much less pronounced and shifted to larger *B*_*z*_ on the chain atoms. The shift again manifests the significant magnetic interactions between the Fe atoms in the chain. Note, that small differences in the spectra between the two inner and between the two outer atoms again result from the slight asymmetry in the chain due to negligible residual couplings to the neighboring chains (see Supplementary Fig. 1). For a further analysis of the spin state of the chain, we quantify the spectra utilizing a perturbation theory model^29^ based on an effective spin Hamiltonian that considers the Zeeman energy, the easy-plane magnetic anisotropy energy, and the Heisenberg as well as DM interactions (see 'Methods'). Astonishingly, by simply considering the atomic parameters known from single hcp Fe atoms on Pt(111)^26^ and using the above Heisenberg and DM interactions extracted from the pairs^25^ as NN interactions in the model (see the parameters in the caption of Fig. 2), we can already excellently reproduce the evolution of the excitation in the ISTS data (cf. Fig. 2c, d). The spectra are rather sensitive to changes in *J*_NN_ and *D*_⊥,NN_, and neither *J*_NN_ nor *D*_⊥,NN_ alone can satisfactorily reproduce the data (see Supplementary Fig. 6). This result further corroborates that the pairwise interaction parameters describe the chain data fairly well. However, for this particular distance, we found that the calculated spectra for the Fe_2_ pair and the Fe_3_ and Fe_4_ chains fit somewhat better to the experimental data when the pairwise Heisenberg coupling^25^ is slightly reduced to *J*_NN_ = −25 µeV, which is justified as the coupling parameters in chains can slightly deviate from the ones in the according pairs^19^. This value was therefore used in all calculations for the *d*_NN_ = 4*a* chains. The corresponding spin-expectation values calculated from exact diagonalization (ED, see 'Methods'), which are illustrated in Fig. 2a reveal that, as long as *B*_*z*_ is not too large, the underlying spin state is highly non-collinear. The spins of neighboring atoms are mutually canted within the (||, *z*)-plane as imposed by the strong *D*_⊥,NN_ component. Note, that a pure spin-spiral state is not realized because of the homogeneous *B*_*z*_ driving all spins into *z*-direction (see Supplementary Note 4 and Supplementary Figs. 11–13). We will later see, how we can avoid this problem by using an RKKY coupling of the chain end to a ferromagnetic island.

### Non-collinear spin states in 3*a* chains

**Fig. 3:**
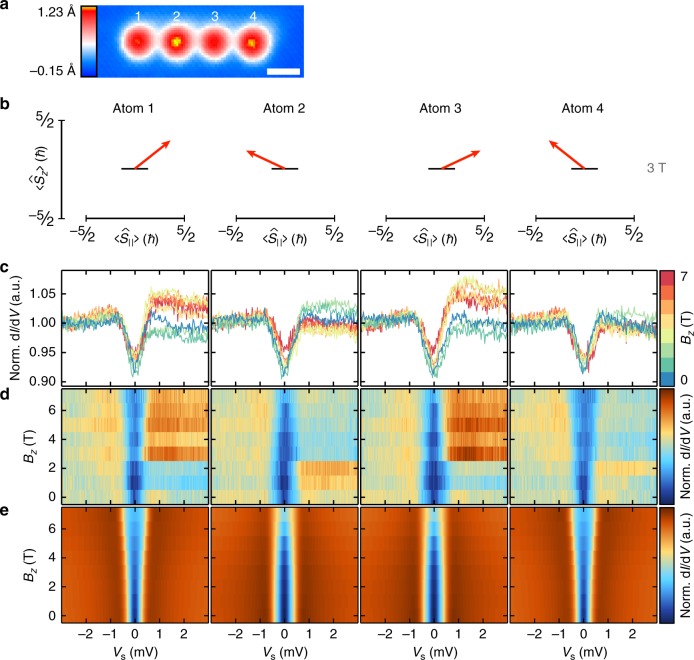
Non-collinear spin state in the Fe_4_ chain with *d*_NN_ = 3*a*. **a** SPSTM topograph of the Fe_4_ chain measured at *B*_*z*_ = 3 T (*V*_s_ = −6 mV, *I*_stab_ = 0.5 nA). The white scale bar has a length of 1 nm. **b** Calculated spin-expectation values at *B*_*z*_ = 3 T from ED for each atom in the Fe_4_ chain, using the parameters given in Table 1, and *J*_NN_ = −60 μeV, *D*_⊥,NN_ = −50 μeV, *J*_NNN_ = +15 μeV, and *D*_⊥,NNN_ = −20 μeV. **c**, **d** ISTS spectra taken with a magnetic tip (SP-ISTS, **c**) and corresponding colorplots (**d**) measured on all four atoms in the Fe_4_ chain in a magnetic field up to *B*_*z*_ = 7 T. (*V*_stab_ = −6 mV, *I*_stab_ = 3 nA, *V*_mod_ = 40 μV). **e** Calculated ISTS from the perturbation theory model using the same parameters as in (**b**)

Next, in order to demonstrate the adjustability of the non-collinear chain state by *d*_NN_, we investigated Fe_*N*_ chains (*N* = 2, 3, 4) with smaller *d*_NN_ = 3*a* (see the ISTS data and calculations of Fe_4_ in Fig. 3, all other chains are shown in Supplementary Figs. 7–10 as described in Supplementary Note 3 again showing a reproducible evolution of the spectra from *N* = 2 to 4). The smaller *d*_NN_ induces a stronger antiferromagnetic Heisenberg component (*J*_NN_ ≈ −60 µeV) and a stronger DM component (*D*_⊥,NN_ ≈ −50 μeV) in pairs^25^ as compared to the 4*a*-case studied above. Most notably, we know from ab initio calculations of the same pairs, that the sign of *D*_⊥,NN_ is reversed^25^. Indeed, *B*_*z*_ dependent ISTS of this Fe_4_ chain behaves rather differently compared to the 4*a*-chain data (cf. Figs. 2b, c and 3c, d): (i) the zero field spin-excitation appears at a larger energy; (ii) for the two inner atoms, the shift of the spin-excitation to lower energies continues here up to at least *B*_*z*_ = 7 T; (iii) for the two end atoms, the excitation energy monotonously increases. These differences can be assigned to the change in *J*_NN_ and *D*_⊥,NN_, as supported by the perturbation theory model calculations of the spectra shown in Fig. 3e, which nicely reproduce the data. Again, we simply used the NN interactions extracted from the according pair with *d* = 3*a*, but additionally took into account the next nearest neighbor (NNN) interactions extracted from the couplings of a pair with *d* = 6*a*^25^ (see the values given in the caption of Fig. 3). As corroborated by ED, the underlying spin state of the chain stabilized in *B*_*z*_ = 3 T (see Fig. 3b) is again strongly non-collinear.

Moreover, the character of the two non-collinear spin states of the 3*a*- and 4*a*-chains is obviously different (cf. Fig. 2a–3b). While the spins of the 1st and 3rd (2nd and 4th) atom in the 4*a*-chain are tilted to the left (right) (cf. Fig. 2a), this tilt is opposite for the 3*a*-chain (cf. Fig. 3b). This is a result of the sign change of *D*_⊥,NN_ between the two cases, which will lead to a different rotational sense if a pure spin-spiral state was stabilized. We finally allude to a subtle difference between the experimental (Fig. 3d) and calculated (Fig. 3e) ISTS. While the conductance levels at positive and negative bias above the excitation threshold are the same in the calculations, there is a systematic difference in the experimental data. For *B*_*z*_ < 2 T the positive-bias conductance is lower (higher) for the 1st and 3rd (2nd and 4th) atom, and this contrast is reversed at *B*_*z*_ = 3 T. Considering the alternating tilts of the spins in the ||-direction (cf. Fig. 3b), we assign this asymmetry to a non-zero ||-component of the spin-polarization of this particular tip, which is reversing at *B*_*z*_ = 3 T. This conclusion is further supported by the alternating heights of neighboring atoms in the SPSTM image taken on this Fe_4_ chain (Fig. 3a). Note, that the spin-polarization is not taken into account in the calculation of the ISTS data.

### Spin-spiral state in a 16 atom 3*a* chain

**Fig. 4:**
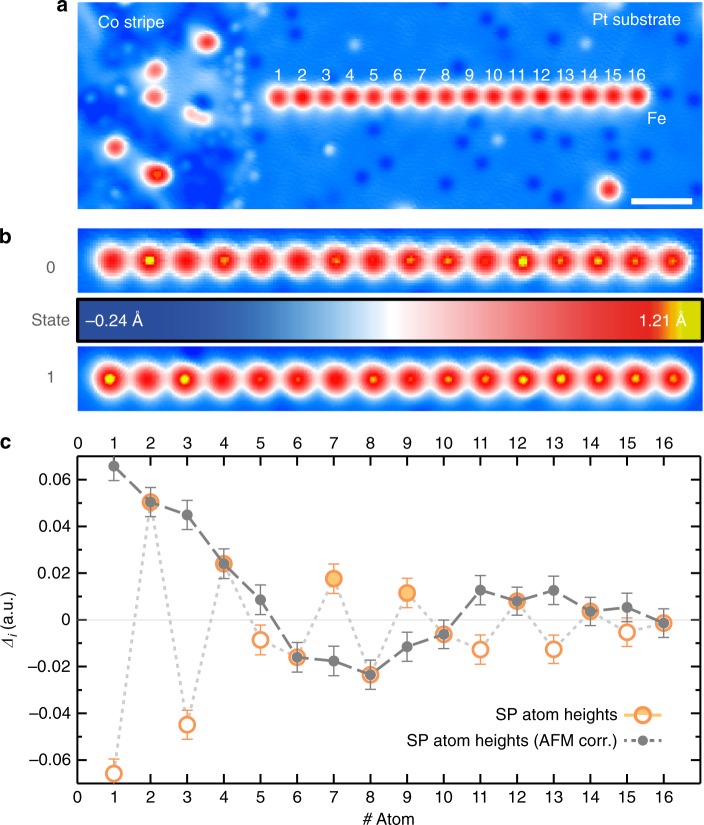
Spin-spiral in Fe_16_ chain coupled to a Co stripe. **a** STM topograph of an Fe_16_ chain with *d*_NN_ = 3*a* RKKY-coupled to a ferromagnetic Co stripe on the left. (*I*_s_ = 0.5 nA, *V*_s_ = 6 mV). The white scale bar has a length of 2 nm. **b** SPSTM images of the two states **0** and **1** of the Fe_16_ chain stabilized by the two magnetization states of the Co stripe taken with out-of-plane sensitive tip (*B*_*z*_ = 0 T, *I*_s_ = 0.5 nA, *V*_s_ = 6 mV). The magnetic state of the Co stripe was reversed between top and bottom image. **c** Spin-asymmetry *Δ*_*i*_ between states **1** and **0** of atom *i* of the Fe_16_ chain taken from the images in **b** (see text). For the gray points, the short-range antiferromagnetic component has been removed by reversing the sign of all even numbered atoms, such that the long range spin-spiral component is visible. The error bars were determined via error propagation from the standard deviation of each measured height (measured three times)

In order to stabilize the pure spin-spiral state we have built a 3*a*-chain with one end RKKY-coupled to a ferromagnetic Co stripe (Fig. 4). The Co stripe has a remanent out-of-plane magnetization, which can be used to stabilize the magnetization of atoms that are adsorbed on the Pt(111) at several nanometers lateral distance^27^. The first atom of the chain was put at a lateral distance of *d*_Co−Fe_ ≈ 1.5 nm to the stripe, where the interaction showed strongest antiferromagnetic coupling^27^. Starting with this atom, an Fe_16_ chain with *d*_NN_ = 3*a* aligned almost perpendicular to the rim of the Co stripe has been assembled (Fig. 4a). Since the RKKY-coupling to the stripe decays rather slowly with lateral distance, we expect possible residual couplings to the 2nd and 3rd chain atoms. However, for all other chain atoms the RKKY interaction to the Co stripe is already an order of magnitude smaller than the mutual RKKY interaction between the atoms within the chain (cp. ^27^. and ^25^). We therefore anticipate that a largely undisturbed spin-spiral can form on the chain. The SPSTM image of the chain (Fig. 4b, top panel) taken with a tip with sensitivity to the out-of-plane component of the magnetization (Supplementary Note 5 and Supplementary Fig. 14) reveals an alternating height of neighboring atoms in some areas (cf. atoms 2 to 4, 7 to 9, and 11 to 14) and almost vanishing contrast in between (cf. atoms 5 to 6 and 9 to 11). To prove the magnetic origin of these height differences, the out-of-plane magnetization of the Co stripe was reversed by ramping the field *B*_*z*_ to −1.7 T and back to zero, while the tip was retracted. Due to the RKKY-stabilization, this procedure reverses the magnetization of atom 1 in the chain, and thereby supposedly switches the chain from a state **0** to a state **1** with a reversed magnetization of all chain atoms. Please note that the magnetic state of the tip thereby remained unchanged (Supplementary Note 5 and Supplementary Fig. 14). Indeed, in the subsequently taken SPSTM image of state **1** (Fig. 4b, bottom panel) the alternating height contrast is reversed with respect to state **0**, while the vanishing contrast regions remain unaffected. For a quantitative analysis of the magnetic contrast, we measure the height on top of each atom (*i*) in state **1** (*h*_*i*,1_) and **0** (*h*_*i*,0_) (after correcting the slope of the image by plane subtraction) and calculate the spin-asymmetry *Δ*_*i*_ by *h*_*i*,0_ − *h*_*i*,1_ divided by the sum *h*_*i*,0_ + *h*_*i*,1_, which is directly proportional to the spin-polarization of the atoms along the magnetic orientation of the tip^30^. In Fig. 4c, *Δ*_*i*_ reveals a long-range beating with a wavelength of about 10 atoms superimposed on the short-range antiferromagnetic component (see the gray points in Fig. 4c for which the antiferromagnetic component was removed by reversing the sign of all even numbered atoms). This strongly indicates the stabilization of an antiferromagnetic spin-spiral in the Fe_16_ chain.

### Comparison to DMRG calculations

**Fig. 5:**
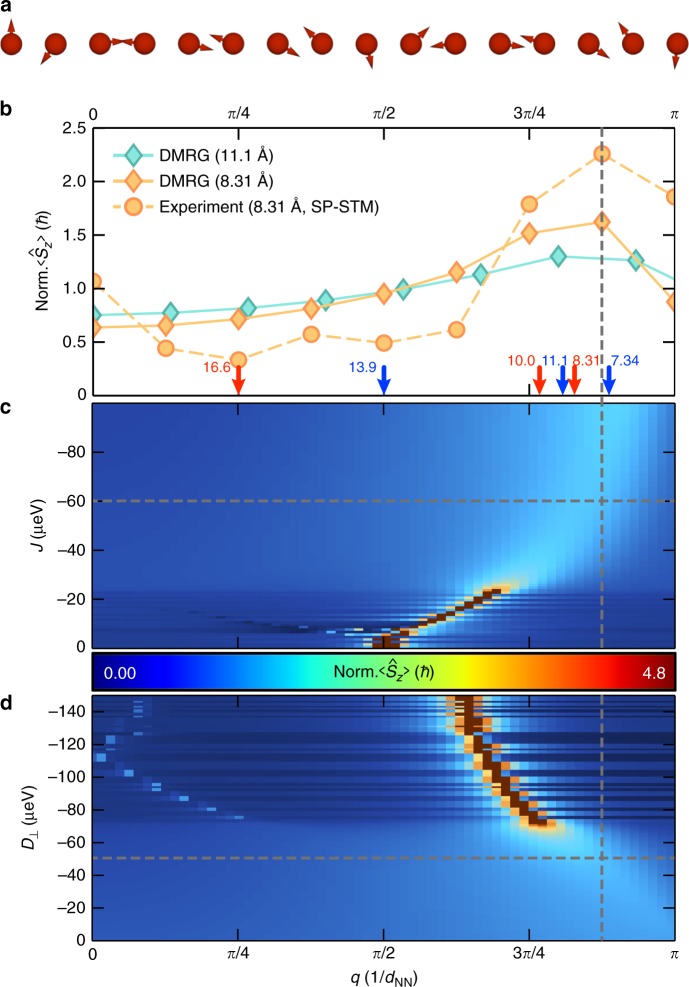
Determination of the spin-spiral wavevector from experiment and DMRG. **a** Illustration of the spin-spiral realized in the 3*a*-chain using the DMRG calculated vectors $( {\langle {\hat S_{||,i}} \rangle ,\langle {\hat S_{z,i}} \rangle } )$ with the length set to unity. **b** Comparison of the Fourier transform of the experimental spin-asymmetry (orange dots) taken from Fig. 4 with the spin-structure factor $\langle {\hat S_z( q )} \rangle$ obtained from the DMRG calculation of a *d*_NN_ = 3*a* (*N* = 16, orange diamonds) and a *d*_NN_ = 4*a* (*N* = 16, cyan diamonds) chain using the NN interactions experimentally determined from pairs (*d*_NN_ = 3*a*: *J*_NN_ = −60 μeV, *D*_⊥,NN_ = −50 μeV; *d*_NN_ = 4*a*: *J*_NN_ = −25 μeV, *D*_⊥,NN_ = +30 μeV; remaining parameters given in Table 1). The curves are normalized to the area. The colored arrows indicate the calculated wavevectors *q* and rotational sense (blue: positive, red: negative) of spin-spirals in chains (*N* = 128) with *d*_NN_ given in Å by the numbers besides the arrows. They were determined from the maxima of the calculated $\langle {\hat S_z( q )} \rangle$ using the experimental pair-interactions from ref. ^25^ (slightly adjusted *J*_NN_ for *d*_NN_ = 4*a*, see Supplementary Fig. 16). **c** $\langle {\hat S_z( q )} \rangle$ as a function of *J* with a constant *D*_⊥_ = −50 μeV (*N* = 128). **d** $\langle {\hat S_z( q )} \rangle$ as a function of *D*_⊥_ with a constant *J* = −60 μeV (*N* = 128). The experimental parameters of the 3*a*-chain are indicated by the dashed gray line

This finding is further corroborated in Fig. 5b by a comparison of the Fourier transform of the measured spin-asymmetry *Δ*_*i*_ with the *z*-component of the spin-structure factor $\left\langle {\hat S_z\left( q \right)} \right\rangle$ obtained from a DMRG calculation for an isolated spin *S* = 5/2 chain with the first spin pinned along *z* (see 'Methods'). We used the same NN coupling parameters as for the ED and perturbation theory model calculations, which were extracted from the experimental investigation of pairs^25^. The experimental and theoretical curves show a very similar trend with the formation of a clear maximum at the wavevector *q* ≈ 7*π*/(8*d*_NN_) (see the dashed vertical line). This finally substantiates the formation of a spin-spiral in the bottom-up constructed Fe_16_ chain with a wavelength of *λ* = 2*π*/*q* ≈ 2.29*d*_NN_ = 19 Å, which is shown in real space in Fig. 5a. The commensuration length of this spin-spiral resulting from *λ* is approximately 7 atoms.

## Discussion

We finally discuss the tunability of the spin-spiral period via the exchange constant ratio *r* = |*D*_⊥,NN_|/|*J*_NN_|, which can be tailored by the distance dependent RKKY-interaction as shown above. Figure 5c, d illustrates the DMRG calculation of $\left\langle {\hat S_z\left( q \right)} \right\rangle$ for long chains (*N* = 128) as a function of *r* while keeping *D*_⊥,NN_ (c) or *J*_NN_ (d) constant, revealing how *q* depends on *r*. *q* approaches *π*/(2*d*_NN_) for large *r*, corresponding to a spin-spiral with perpendicular orientations of neighboring spins. For *r* ≈ 1, which corresponds to the case experimentally realized here, the peak gets broader due to the competition of Heisenberg and DM interactions, and the magnetic anisotropy, but is still well defined, indicating that the wavevector of the antiferromagnetic (*J*_NN_ < 0) spin-spiral can in principle be adjusted within the range *π*/(2*d*_NN_) < *q* < *π*/*d*_NN_. For the other experimentally realized 4*a*-chain, the DMRG calculated $\langle {\hat S_z( q )} \rangle$ (see cyan curve in Fig. 5b) predicts a maximum at *q* ≈ 104*π*/(128*d*_NN_) corresponding to an antiferromagnetic spin-spiral with a larger wavelength of *λ* ≈ 27 Å and opposite rotational sense as compared to the 3*a*-chain.

Summarizing, we demonstrated that the rotational sense and the period of spin-spirals realized in bottom-up constructed chains of RKKY-coupled transition-metal atoms on a high-*Z* substrate can be tuned by the NN distance of the chain atoms. Building chains with other experimentally accessible NN distances will enable to further adjust *J*_NN_ and *D*_⊥,NN_^25^, including ferromagnetic Heisenberg interaction. Our DMRG calculations of according chains using the experimentally determined couplings^25^ predict that *q* (see the colored arrows in Fig. 5b, Supplementary Note 7 with Supplementary Fig. 16 and Supplementary Table 1) can thereby be varied from *π*/(4*d*_NN_) to 114*π*/(128*d*_NN_), corresponding to a large variation in the wavelength from *λ* ≈ 17 Å to 133 Å. This tunability of non-collinear spin states could be very important for the manipulation of Majorana end modes of such chains assembled on a superconducting substrate^12,13^.

## Methods

### Experimental procedures

All experiments were performed in a home-built STM facility^31^ at a temperature of 0.3 K. A magnetic field of up to *B*_*z*_ = 12 T was applied perpendicular to the sample surface. The Pt(111) sample was cleaned in situ by subsequent sputter-and-anneal cycles as well as O_2_ annealing and high temperature flashes^25,26^. Finally the sample was flashed to 1000 °C for 1 min. In order to prepare the Co stripes, a fraction of a monolayer Co was deposited from an electron-beam heated rod during cooling down the sample to room temperature after the final flash, resulting in the decoration of the Pt step edges and terraces with one atomic layer high Co stripes and islands, respectively^27^. For the deposition of Fe atoms, about 1% of a monolayer Fe was evaporated onto the cold sample kept at $T \lesssim 10\,{\mathrm{K}}$.

STM topographs were recorded in the constant-current mode of the STM at a stabilization current *I*_stab_ and voltage *V*_s_ applied to the sample. For the assembly of the Fe chains, the Fe atoms were manipulated using lateral tip-induced atom manipulation^32^ with a current of *I*_stab_ ≈ 40 nA and a voltage of *V*_s_ = 2 mV. We used flashed and Cr coated (≈50 ML) tungsten tips which were prepared as described before^32^. In order to achieve magnetic contrast in the SPSTM images or in SP-ISTS with a sensitivity to the out-of-plane component of the sample magnetization (along *z*), the tip was dipped up to 500 pm into a Co layer and several single Fe atoms were picked up, until a sufficient contrast was observed on the remanently out-of-plane magnetized Co stripes or islands (see Supplementary Fig. 14). For non-magnetic ISTS measurements, voltage pulses were applied to the tip and/or it was gently dipped into the Pt substrate until the magnetic contrast vanished and a spectroscopically flat Pt substrate spectrum within the voltage range used for ISTS was obtained^32^. ISTS was performed by adding a modulation voltage *V*_mod_ (rms) of frequency *f*_mod_ = 4.142 kHz to *V*_s_. After stabilizing the tip at *I*_stab_ and switching the feedback off, the bias *V*_s_ was ramped from the start to the end voltage of the desired spectroscopy range. Simultaneously, the differential conductance d*I/*d*V* was recorded via a lock-in amplifier. In order to eliminate tip related features, all presented spectra of atoms were normalized by dividing by a spectrum taken with the identical tip on the bare Pt substrate.

### Perturbation theory model

The experimental ISTS data was modeled by employing the perturbation theory code (v0.999) by Ternes^29^. The model is based on the following effective spin Hamiltonian

1 $$\begin{array}{*{20}{l}} {\hat {\cal H}} \hfill & = \hfill & {\hat {\cal H}_{{\mathrm{Zeeman}}} + \hat {\cal H}_{{\mathrm{anisotropy}}} + \hat {\cal H}_{{\mathrm{Heisenberg}}} + \hat {\cal H}_{{\mathrm{DM}}}} \hfill \\ {} \hfill & = \hfill & { - \mathop {\sum}\limits_i^N g\mu _{\mathrm{B}}{\mathbf{B}} \cdot {\hat{\mathbf S}}_i + \mathop {\sum}\limits_i^N K\left( {\hat S_{i,z}} \right)^{\mathrm{2}}} \hfill \\ {} \hfill & {} \hfill & { - \mathop {\sum}\limits_{i \ne j}^N J_{ij}{\kern 1pt} {\hat{\mathbf S}}_i \cdot {\hat{\mathbf S}}_j - \mathop {\sum}\limits_{i \ne j}^N {\mathbf{D}}_{ij}\left( {{\hat{\mathbf S}}_{\mathrm{i}} \times {\hat{\mathbf S}}_{\mathrm{j}}} \right),} \hfill \end{array}$$

which includes the Zeeman energy and the magnetic anisotropy of all atoms in the chain, quantified by the Landé g-factor *g*, Bohr magneton *μ*_B_, and axial magnetic anisotropy constant *K*, respectively, as well as the mutual Heisenberg and the DM interactions between atoms *i* and *j* quantified by the Heisenberg exchange constant *J*_*ij*_ and DM vector **D**_*ij*_, respectively. Note, that no third order effects had to be considered to nicely reproduce the data. All model parameters which are not specified in the text were kept constant and are given in Table 1 (same parameters as used in Ref.^25^).

**Table 1:** Simulation parameters

	*S*	*g*	*K* (in meV)	*U*	*Jρ*_0_	*ω* (in meV)	*T*_eff_ (in K)
	5/2	2.0	+0.08	0	−0.02	20	0.6

Parameters used for all simulations of experimental data shown in this article

### ED and DMRG calculations

To solve the model Equation 1 for the *S* = 5/2 chains up to *N* = 4, we used ED. For the longer chains (*N* = 16 and *N* = 128) we used our DMRG code^33^. Note, that the Hilbert space of 16 atoms becomes prohibitively large since we are dealing with a spin *S* = 5/2. The DMRG method exploits the relatively small entanglement between parts of the system to compress the wavefunction into a matrix product state which is controlled by the bond dimension *χ* (a measure for the amount of variational parameters), and yields essentially exact results. The summations are restricted to NN.

Our algorithm adaptively chooses the bond dimension *χ* using the energy variance $\varepsilon = \left| {\left\langle {\hat {\cal H}^2} \right\rangle - \left\langle {\hat {\cal H}} \right\rangle }^2 \right|/N$ as a convergence criterion for the ground state (in addition to the ground-state energy), where $\varepsilon < 10^{ - 6}$ can be typically achieved with *χ* ~ 50–100 for a system of *N* = 128 sites. Note, that a well-defined peak in $\left\langle {\hat S_z\left( q \right)} \right\rangle$ is already obtained for *N* = 16, which does not change significantly for *N* = 128 sites (cf. Supplementary Fig. 15 and Supplementary Note 6).

Note, that the DM term in Equation 1 breaks the SU(2) symmetry of the Heisenberg model, such that all the components $\left\langle {\hat S_{||,i}} \right\rangle$, $\left\langle {\hat S_{ \bot ,i}} \right\rangle$ and $\left\langle {\hat S_{z,i}} \right\rangle$ are in general nonvanishing and the Hamiltonian is complex. To determine the wavevector *q* of the spiral in a chain of length *N*, we calculate $\left| {\left\langle {\hat S_\alpha } \right\rangle \left( q \right)} \right| = 1/N\left| {\mathop {\sum}\nolimits_{n = 0}^{N - 1} \left\langle {\hat S_{\alpha ,n}} \right\rangle e^{{\mathrm{i}}qn}} \right|$ with $\alpha = ||, \bot ,z$ and *q* taking the values $q_n = 2\pi n/N$, where $n = 0,1, \ldots ,N - 1$. For $D_{||} = 0$ and $D_z = 0$ the Hamiltonian becomes real, which speeds up the calculations considerably. Furthermore, the ground state can be chosen real as well, meaning that the perpendicular component vanishes: $\left\langle {\hat S_{ \bot ,i}} \right\rangle = {\mathrm{Im}}\left\langle {\hat S_i^ + } \right\rangle = 0$.

### Data availability

The authors declare that all relevant data are included in the paper and its Supplementary Information files. Additional data are available from the corresponding author upon reasonable request. Some references concerning data analysis and display are refs.^34,35^.

## Electronic supplementary material

Supplementary Information
