# Author Correction: Non-collinear spin states in bottom-up fabricated atomic chains

**DOI:** 10.1038/s41467-018-07428-y

**Published:** 2018-11-19

**Authors:** Manuel Steinbrecher, Roman Rausch, Khai Ton That, Jan Hermenau, Alexander A. Khajetoorians, Michael Potthoff, Roland Wiesendanger, Jens Wiebe

**Affiliations:** 1 Department of Physics, Hamburg University, Jungiusstrasse 9A, 20355 Hamburg, Germany; 2 Institute for Molecules and Materials (IMM), Radboud University, P.O. Box 9010//078, 6500 GL Nijmegen, The Netherlands; 3 I. Institute for Theoretical Physics, Hamburg University, Jungiusstrasse 9, 20355 Hamburg, Germany

Correction to: *Nature Communications* 10.1038/s41467-018-05364-5; published online 20 July 2018

The original version of this Article contained an error in Fig. 3 in which Fig. 3b and Fig. 3e incorrectly duplicated Fig. 2a and Fig. 2d, respectively. This has now been corrected in both the PDF and HTML versions of the Article.

