# Supplementary material for: Non-collinear spin states in bottom-up fabricated atomic chains
Source: Nat Commun. 2018 Jul 20;9:2853. doi: 10.1038/s41467-018-05364-5 (PMC6054618; doi:10.1038/s41467-018-05364-5)
Supplement: Supplementary file 1 — Supplementary Information [file 41467_2018_5364_MOESM1_ESM.pdf]

Non-collinear spin states in bottom-up fabricated atomic chains

Steinbrecher *et al.*

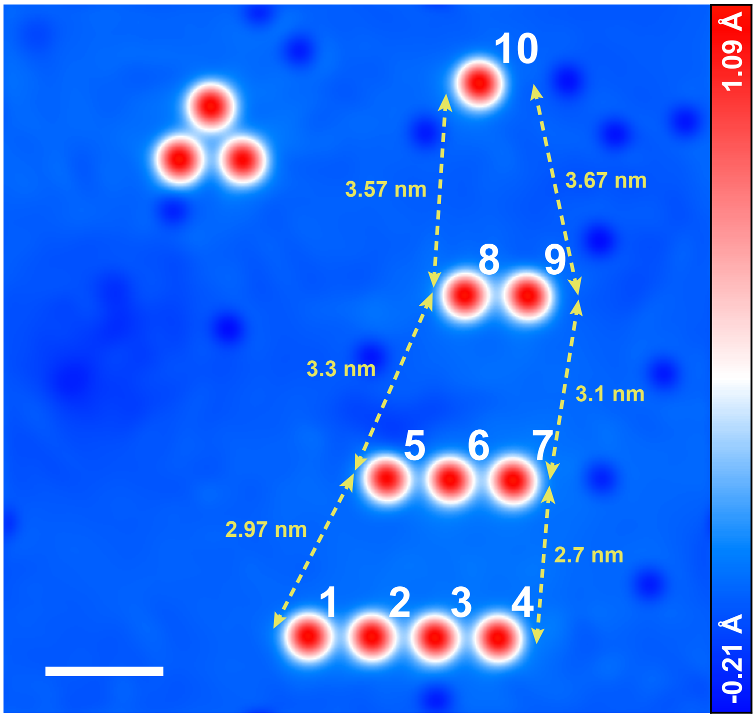

Supplementary Figure 1 | Overview of the 4*a* chain array. The chains were built with several nanometer separation (as indicated, the white scale bar has a length of $2\,\mathrm{nm}$) in order to minimize mutual interactions between the chains ($V_\mathrm{s} = -6\,\mathrm{mV}$, $I_\mathrm{stab} = 0.5\,\mathrm{nA}$). Nevertheless, the two end atoms in each chain are not completely equivalent considering their distances to the atoms of the neighboring chains: the left end atom in each chain has a slightly larger distance to the atoms of the neighboring chains than the right end atom. Taking into account the residual magnetic interactions between neighboring chains, this leads to a tiny residual shift of the excitation energy which is different for the two end atoms, as seen in Figure 1a of the main manuscript. This effect is more pronounced for the Fe_4 (atoms 1-4) and Fe_3 (atoms 5-7) chains as compared to the Fe_2 (atoms 8,9) pair, as they have a smaller separation. However, note, that these residual couplings are negligible as compared to the couplings within each chain, and will therefore not affect our conclusions on the non-collinear spin states.

Supplementary Note 1 | Labeling and coordinate system

In order to clarify the coordinate system used within the main manuscript text, a sketch of all directions and vector components is shown in Supplementary Figure 1. Here, a chain built from three Fe atoms is placed on top of the Pt(111) surface with a mutual distance of three lattice sites (8.31 Å). The gray coordinate system on the top right defines the labeling of the components of all vectors mentioned.

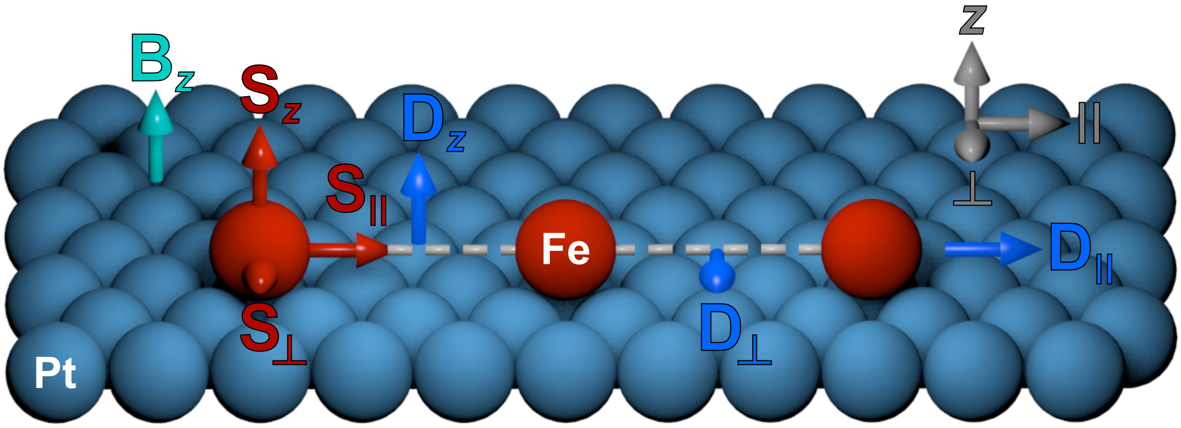

Supplementary Figure 2 | Sketch of directions. The schematic ball model shows a chain consisting of three Fe atoms on a Pt(111) surface to explain the used nomenclature. The different directions are defined by the gray coordinate system and are adapted from a previous publication[1]. The components of the magnetic field $\mathbf{B}$, the spin $\mathbf{S}$ and the Dzyaloshinskii-Moriya vector $\mathbf{D}$ are labeled accordingly.

Supplementary Note 2 | Additional ISTS measurements of 4*a* chains

In addition to the measurements of the Fe_4 chain shown in Figure 2 of the main manuscript, the spectra of the remaining 4*a* chains (atoms 5 - 10) are shown in Supplementary Figure 3. The complementary colorplot representation of the spectra is given in Supplementary Figure 4 and the corresponding calculations using third-order perturbation theory[2] are plotted in Supplementary Figure 5. The different effects of the Heisenberg and Dzyaloshinskii-Moriya interaction on the spectra, and that we can indeed distinguish them, is shown in Supplementary Figure 6.

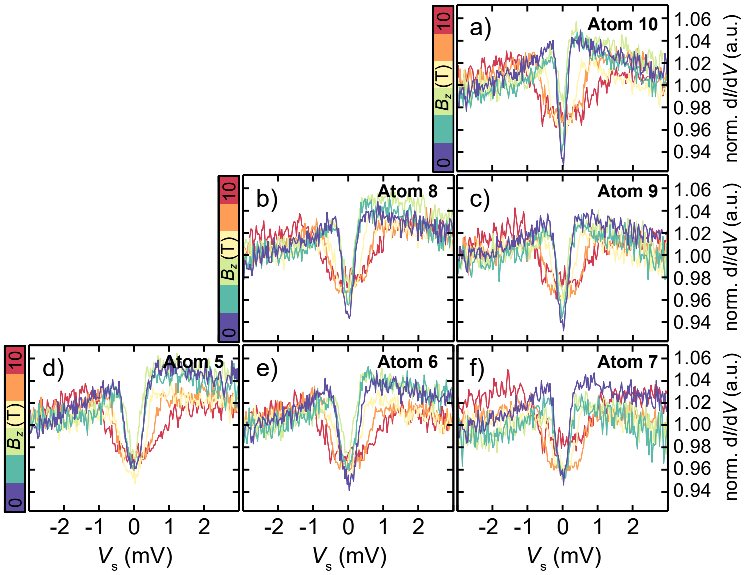

Supplementary Figure 3 | ISTS spectra of all atoms of the 4*a* chains Fe_3, Fe_2, and Fe_1. Fe_3 chain: atoms 5 to 7; Fe_2 chain: atoms 8, 9; Fe_1: atom 10. For clarity, every second spectrum has been omitted in this plot. ($V_{\rm stab} = -6\,{\rm mV}$, $I_{\rm stab} = 3\,{\rm nA}$, $V_{\rm mod} = 40\,\mu{\rm V}$).

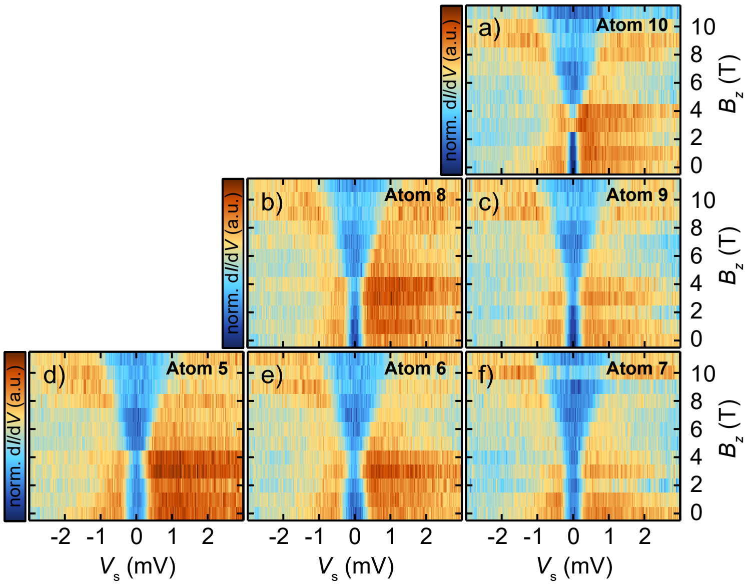

Supplementary Figure 4 | ISTS colorplots of all atoms of the 4*a* chains Fe_3, Fe_2, and Fe_1. In contrast to Supplementary Figure 3, all spectra from $B_z = 0 - 11\,\mathrm{T}$ are given in this plot (Fe_3 chain: atoms 5 to 7; Fe_2 chain: atoms 8, 9; Fe_1: atom 10). This color coding enables an easier recognition of the evolution of the spin-excitation ($V_{\mathrm{stab}} = -6\,\mathrm{mV}$, $I_{\mathrm{stab}} = 3\,\mathrm{nA}$, $V_{\mathrm{mod}} = 40\,\mu\mathrm{V}$).

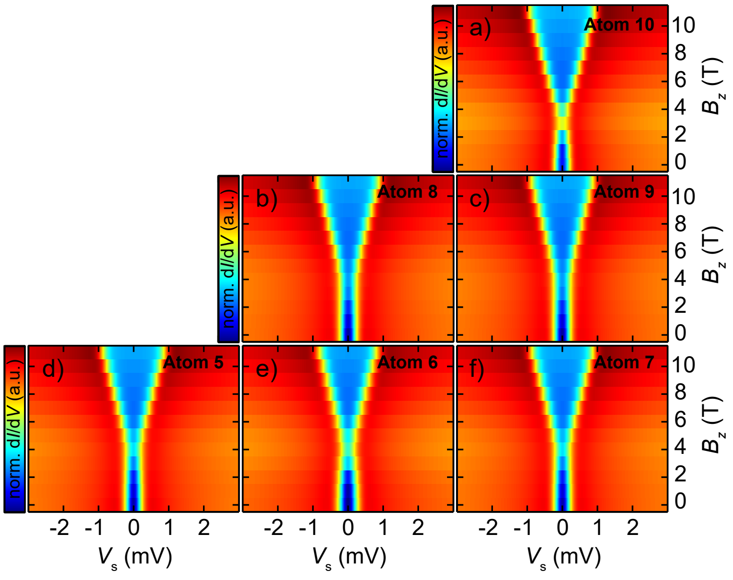

Supplementary Figure 5 | ISTS calculations of all atoms of the 4*a* chains Fe_3, Fe_2, and Fe_1. The perturbation theory model[2] was used to calculate ISTS spectra of all atoms (Fe_3 chain: atoms 5 to 7; Fe_2 chain: atoms 8, 9; Fe_1: atom 10). The used parameters are the same as in Figure 2 of the main manuscript ($J_{\mathrm{NN}} = -25\,\mu\mathrm{eV}$, $D_{\perp,\mathrm{NN}} = 30\,\mu\mathrm{eV}$).

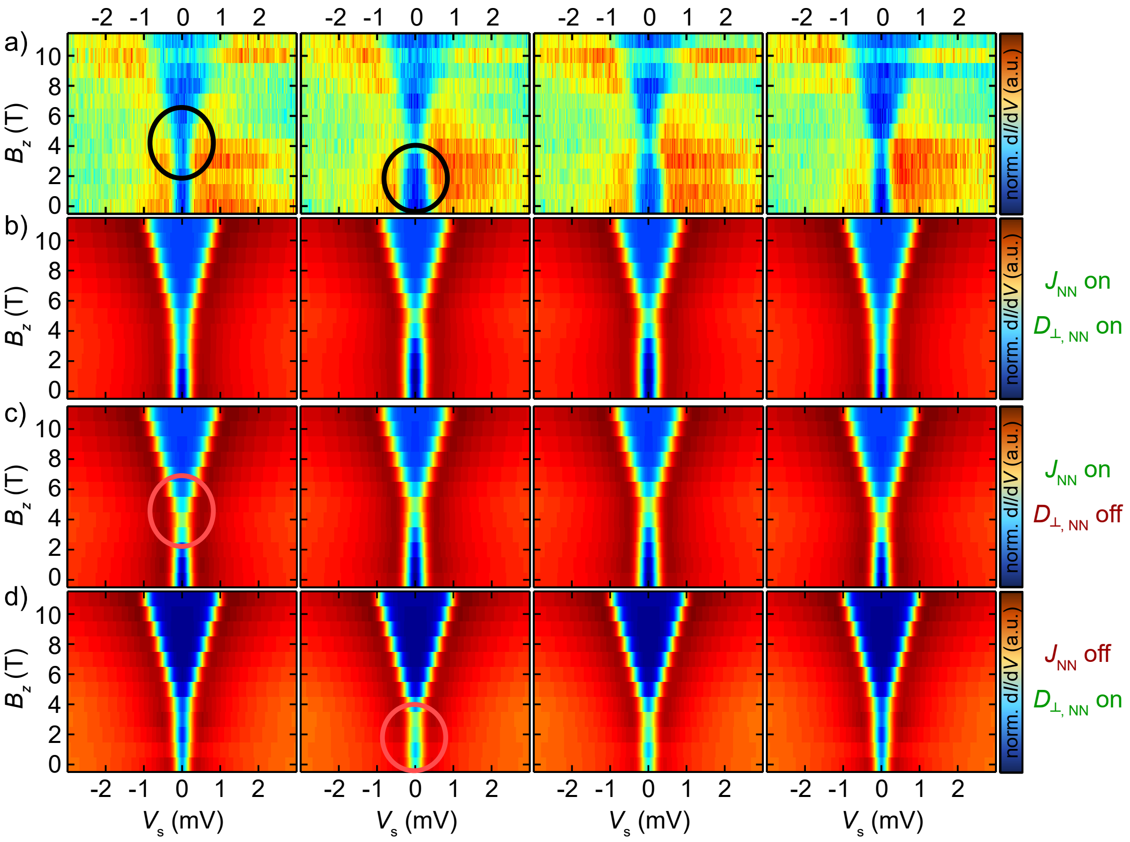

Supplementary Figure 6 | Sensitivity of ISTS spectra to changes in J_{NN} and $D_{\perp,\mathrm{NN}}$. a ISTS spectra of the 4*a* Fe_4 chain (same as Figure 2c, $V_{\mathrm{stab}} = -6\,\mathrm{mV}$, $I_{\mathrm{stab}} = 3\,\mathrm{nA}$, $V_{\mathrm{mod}} = 40\,\mu\mathrm{V}$). **b** Calculated ISTS from the perturbation theory model using the same parameters as in Figure 2 and Table 1 of the main manuscript ($J_{\mathrm{NN}} = -25\,\mu\mathrm{eV}$, $D_{\perp,\mathrm{NN}} = +30\,\mu\mathrm{eV}$). **c**, **d** Same as (**b**), but with $D_{\perp,\mathrm{NN}}$ (**c**) or J_{NN} (**d**) switched off. The red circles mark the most pronounced disagreements with the corresponding features marked by black circles in the experimental data, resulting in the rejection of the according sets of parameters used for (**c**) and (**d**).

Supplementary Note 3 | Additional SPSTM & -ISTS measurements of 3a chains

An STM topograph taken at $3\,\mathrm{T}$ of all investigated $3a$ chains is shown in Supplementary Figure 7. It was recorded with a magnetically sensitive tip resulting in the alternating apparent heights of the atoms, i.e. atoms 2, 4, 5, 7 and 9 are higher than atoms 1, 3, 6 and 8. This height contrast is consistent with the calculated $\langle \hat{S}_{||} \rangle$-component of the atoms' magnetization (Supplementary Figure 12 and 13) assuming a sensitivity of the used tip to the $||$-component of the atom magnetization.

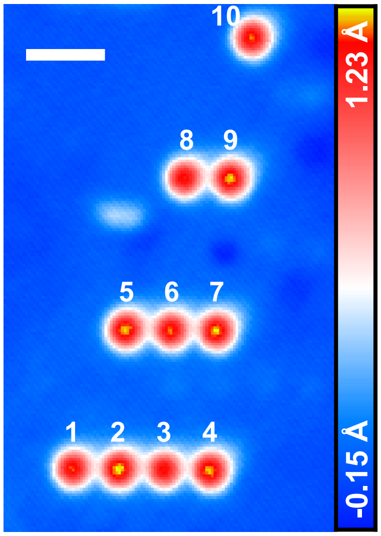

Supplementary Figure 7 | SPSTM image of 3*a* chains. The chains were built with several nanometers separation in order to exclude mutual interactions. Alternating apparent heights are due to the magnetic contrast on the atoms ($V_\mathrm{s} = -6\,\mathrm{mV}$, $I_\mathrm{stab} = 0.5\,\mathrm{nA}$, $B_z = 3\,\mathrm{T}$). The white scale bar has a length of $2\,\mathrm{nm}$.

SP-ISTS experiments were performed on all individual atoms of these chains. While the data taken on the Fe_4 chain is shown in Figure 3 of the main manuscript, the spectra of the remaining chains (atoms 5 - 10) are shown in Supplementary Figure 8. The complementary colorplot representation of the spectra is given in Supplementary Figure 9 and the corresponding results of calculations in the perturbation theory model[2] are plotted in Supplementary Figure 10.

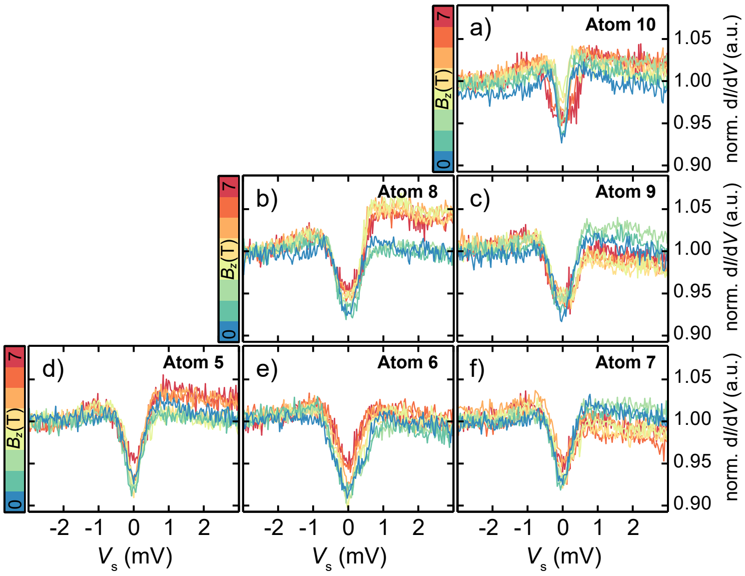

Supplementary Figure 8 | SP-ISTS spectra of all atoms of the 3*a* chains Fe_3, Fe_2, and Fe_1. Fe_3 chain: atoms 5 to 7; Fe_2 chain: atoms 8, 9; Fe_1: atom 10. Magnetic contrast can be recognized by comparing the positive bias conductance levels of atoms 8 and 9 as well as atoms 6 and 7. It results from the sensitivity of the used tip to the $||$-component of the atom magnetization ($V_{\text{stab}} = -6\,\text{mV}$, $I_{\text{stab}} = 3\,\text{nA}$, $V_{\text{mod}} = 40\,\mu\text{V}$).

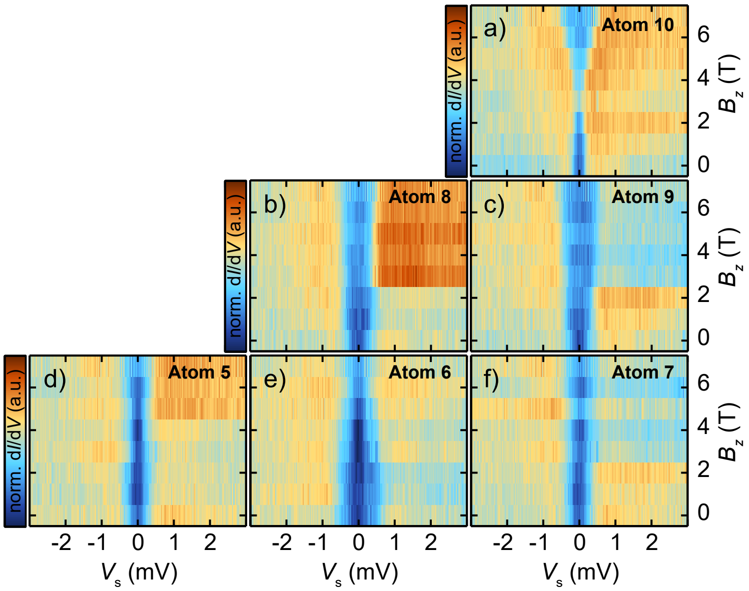

Supplementary Figure 9 | SP-ISTS colorplots of all atoms of the 3*a* chains Fe_3, Fe_2, and Fe_1. Same data as in Supplementary Figure 8, but in the colorplot representation. This enables an easier recognition of the spin-polarized contrast given by the color in the positive bias regime, which alternates from atom to atom in the Fe_3 and Fe_2 chains ($V_{\rm stab} = -6\,\rm mV$, $I_{\rm stab} = 3\,\rm nA$, $V_{\rm mod} = 40\,\mu\rm V$).

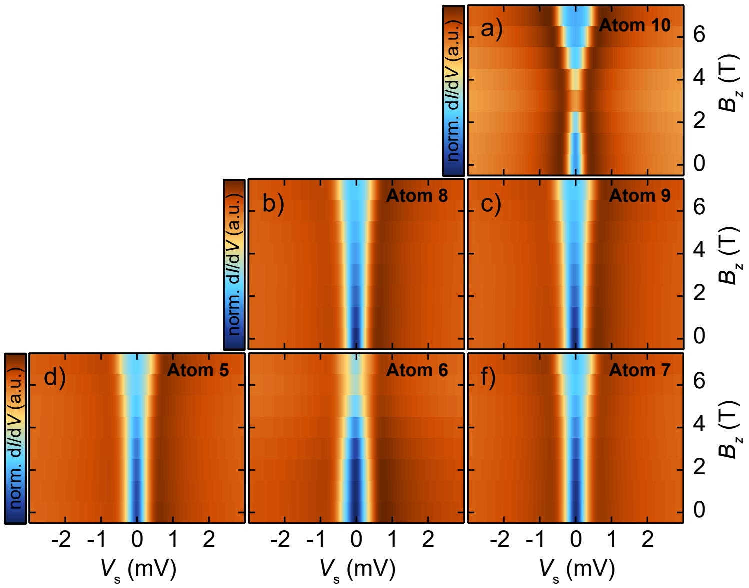

Supplementary Figure 10 | ISTS calculations of all atoms of the 3*a* chains Fe_3, Fe_2, and Fe_1. Perturbation theory model[2] calculation of ISTS of all atoms of the Fe_3 chain (atoms 5 to 7), the Fe_2 chain (atoms 8, 9) and Fe_1 (atom 10). The used parameters are the same as given in Figure 3 of the main manuscript ($J_{\rm NN} = -60\,\mu\rm eV$, $D_{\perp,\rm NN} = -50\,\mu\rm eV$, $J_{\rm NNN} = 15\,\mu\rm eV$ and $D_{\perp,\rm NNN} = -20\,\mu\rm eV$).

Supplementary Note 4 | Calculated magnetization curves

To estimate the states of all individual spins within the investigated chains, magnetization curves were calculated using exact diagonalization (ED) (see methods section in main manuscript). Supplementary Figure 11 shows the results for the Fe_4 chain with $d_{\mathrm{NN}} = 4a$. The pseudo-vectors given in Figure 2a of the main text were calculated from the spin-expectation values at $B_z = 3\,\mathrm{T}$ of these magnetization curves.

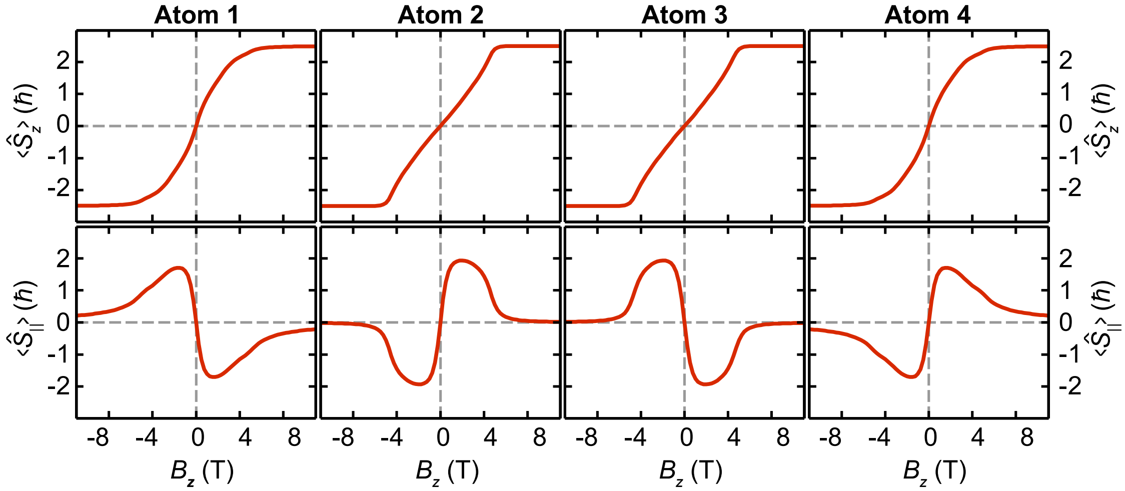

Supplementary Figure 11 | Calculated magnetization curves of Fe_4 chain with $d_{\mathrm{NN}} = 4a$. The expectation values $\langle \hat{S}_z \rangle$ and $\langle \hat{S}_{||} \rangle$ were calculated via ED. By symmetry, $\langle \hat{S}_{\perp} \rangle$ is always zero ($J_{\mathrm{NN}} = -25\,\mu\mathrm{eV}$, $D_{\perp,\mathrm{NN}} = 30\,\mu\mathrm{eV}$).

For the $3a$ chains, the resulting magnetization curves of the Fe_3 and the Fe_4 chain are plotted in Supplementary Figure 12 and 13. Driven by the magnetic field pointing in z-direction, the expectation values $\langle \hat{S}_z \rangle$ of all atoms behave almost the same. Therefore, this component of the spin expectation values cannot be responsible for the alternating magnetic contrast which is observed in the SP-ISTS data in Supplementary Figure 8 and 9 as well as in Figure 3 of the main manuscript. In contrast, $\langle \hat{S}_{||} \rangle$ indeed reveals alternating signs, which can explain the experimentally observed magnetic contrasts. For example, at a positive field of about $5\,\mathrm{T}$, $\langle \hat{S}_{||} \rangle$ of atom 5 is positive, zero for atom 6 and negative for atom 7. This behavior can explain the different conductance levels at positive bias and large magnetic field in Supplementary Figure 9d, e and f if we assume that tip has a sensitivity to the $||$-component of the magnetization. Similarly, in the case of the Fe_4 chain, the sign of $\langle \hat{S}_{||} \rangle$ alternates from atom to atom, which can explain the alternating conductance levels in Figure 3c and d of the main manuscript. We conclude, that the used magnetic tip shows a certain amount of sensitivity to the $||$-component of the magnetization, which enables to image the non-collinear states of the spin chains.

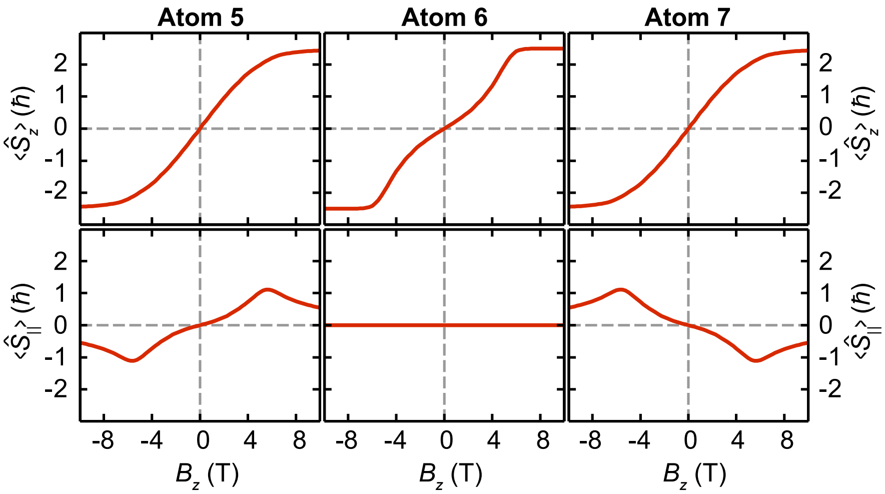

Supplementary Figure 12 | Calculated magnetization curves of Fe_3 chain with $d_{\mathrm{NN}} = 3a$. The expectation values $\langle \hat{S}_z \rangle$ and $\langle \hat{S}_{\parallel} \rangle$ were calculated via ED. By symmetry, $\langle \hat{S}_{\perp} \rangle$ is always zero ($J_{\mathrm{NN}} = -60\,\mu\mathrm{eV}$, $D_{\perp,\mathrm{NN}} = -50\,\mu\mathrm{eV}$, $J_{\mathrm{NNN}} = 15\,\mu\mathrm{eV}$ and $D_{\perp,\mathrm{NNN}} = -20\,\mu\mathrm{eV}$).

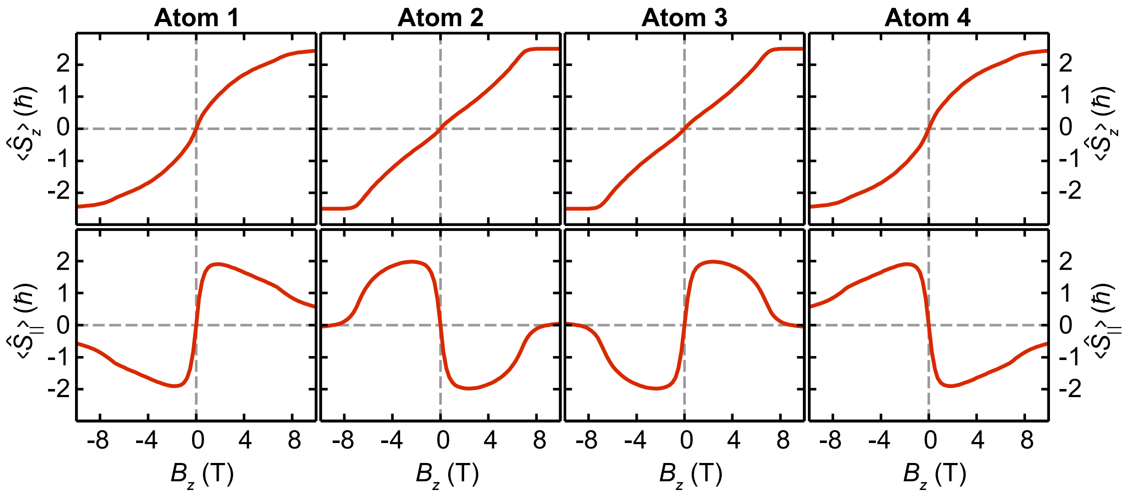

Supplementary Figure 13 | Calculated magnetization curves of Fe_4 chain with $d_{\mathrm{NN}} = 3a$. The expectation values $\langle \hat{S}_z \rangle$ and $\langle \hat{S}_{\parallel} \rangle$ were calculated via ED. By symmetry, $\langle \hat{S}_{\perp} \rangle$ is always zero ($J_{\mathrm{NN}} = -60\,\mu\mathrm{eV}$, $D_{\perp,\mathrm{NN}} = -50\,\mu\mathrm{eV}$, $J_{\mathrm{NNN}} = 15\,\mu\mathrm{eV}$ and $D_{\perp,\mathrm{NNN}} = -20\,\mu\mathrm{eV}$).

Supplementary Note 5 | SPSTS images of the Co stripes

In order to prove that the magnetic tip used for the measurements of Figure 4 of the main manuscript has a sensitivity to the out-of-plane component of the sample magnetization, and that the magnetic orientation of the Co stripe the Fe_{16} chain is coupled to can be reversed, spin-polarized ${}^{\mathrm{d}I}/_{\mathrm{d}V}$ images recorded at $B_z = 0\,\mathrm{T}$ are shown in Supplementary Figure 14. The

imaged area contains two Co stripes (left and right) and the top part of a Co island (middle), which are known to have a remanent magnetization perpendicular to the surface[3]. The Fe_{16} chain is coupled to the left Co stripe, as indicated in the image.

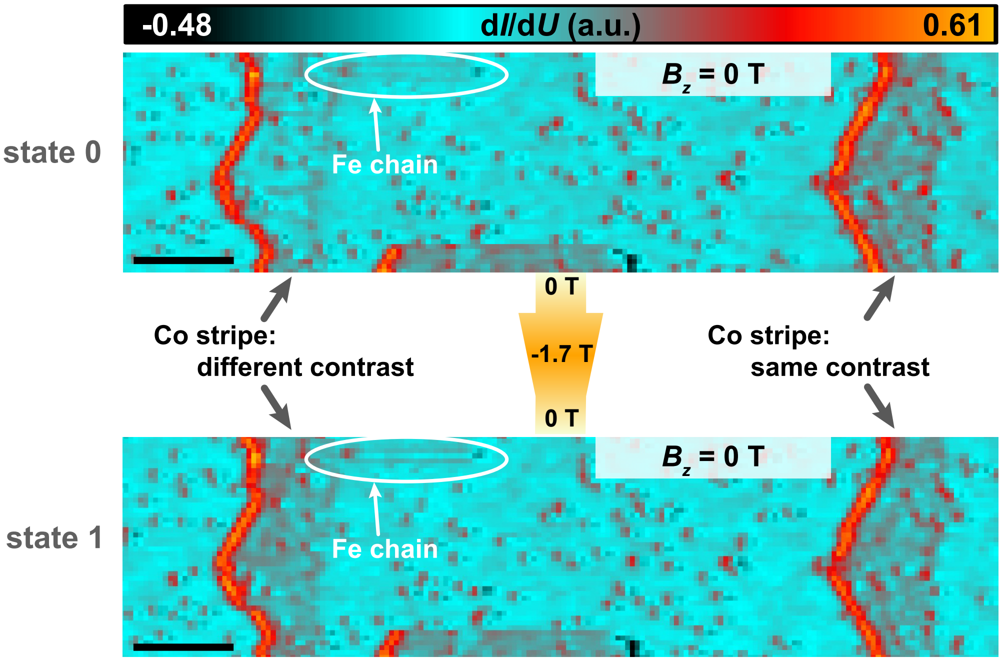

Supplementary Figure 14 | SPSTS images of magnetic Co stripes. Top and bottom images show spin-polarized $^{\mathrm{d}I}/_{\mathrm{d}V}$ maps of an area with two Co stripes and the Fe_{16} chain coupled to the left stripe. In between the images, which were both taken at zero B_z, the magnetic field was ramped to $B_z = -1.7\,\mathrm{T}$ in order to reverse the magnetization of the left stripe ($V_\mathrm{s} = -10\,\mathrm{mV}$, $I_\mathrm{s} = 600\,\mathrm{pA}$, $V_\mathrm{mod} = 5\,\mathrm{mV}$). The black scale bars have a length of $10\,\mathrm{nm}$

In the image on the top, the left Co stripe appears bright, while the right stripe as well as the island appear darker. The contrast difference is ascribed to an opposite out-of-plane magnetization of the left and right stripe imaged by the tip with a sensitivity to the out-of-plane component of the sample magnetization. In the following, we define the magnetization state of the bright stripe as state **0**, and that of the darker stripe as state **1**. Next, the magnetic field is ramped to $B_z = -1.7\,\mathrm{T}$ and back to $0\,\mathrm{T}$. The spin-resolved image recorded afterwards of the same area (bottom image) reveals a dark contrast on both Co stripes and the island. The contrast change from bright to dark of the left stripe is consequently assigned to a reversal of its magnetization to state **1**. Note, that we cannot fully exclude a magnetization reversal of the tip and all magnetic structures but the left stripe, as this would lead to the same behavior of the magnetic contrasts in the images. However, this possibility is rather unlikely. Most importantly, even in this case, the relative orientations of the tip and Co stripe magnetizations between the two images are still reversed. Therefore, the analysis of the data and all conclusions made in the main manuscript remain valid.

Supplementary Note 6 | System-size dependence of the DMRG calculation

Supplementary Figure 15 shows the system-size dependence of the spin structure factor $\langle \hat{S}_z(q) \rangle$, calculated with DMRG and normalized to the area. One observes that a chain length of $N = 16$ used in the experiment is already sufficient for a well-defined peak in $\langle \hat{S}_z(q) \rangle$ that does not change much with increasing size. With $N = 128$, one already obtains the converged result, such that considering larger systems is not necessary. This is due to the expectation values $\langle \hat{S}_{z,i} \rangle$ and $\langle \hat{S}_{\parallel,i} \rangle$ approaching zero for sites i far away from the local B_z-field at the first site.

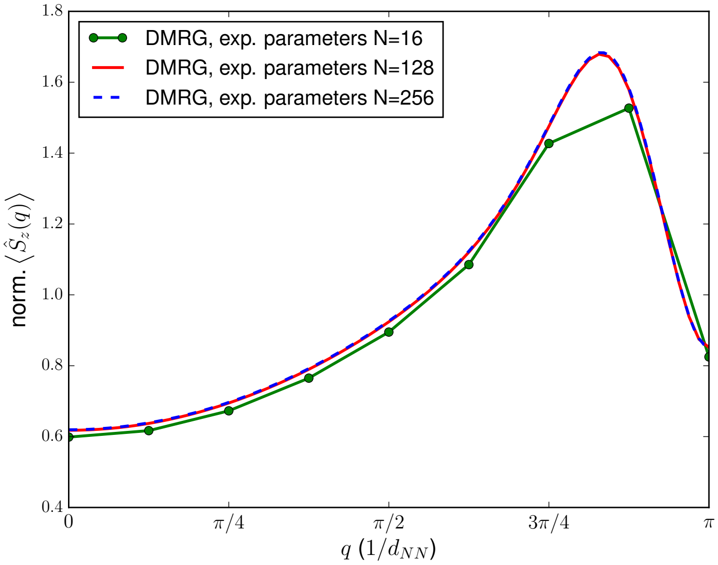

Supplementary Figure 15 | Comparison of the DMRG-calculated spin-structure factor $\langle \hat{S}_z(q) \rangle$ for chains of different numbers N of atoms. The following parameters have been used: $J_{\mathrm{NN}} = -60\,\mu\mathrm{eV}$, $D_{\perp,\mathrm{NN}} = -50\,\mu\mathrm{eV}$, $K = 80\,\mu\mathrm{eV}$.

Supplementary Note 7 | Tuning of the spin-spiral wavelength

Supplementary Figure 16 shows the spin-structure factors $\langle \hat{S}_z(q) \rangle$ calculated for chains with the first spin pinned along z and the different coupling parameters which are experimentally accessible using hcp Fe atoms with the mutual distances d investigated in our previous publication[1]. The coupling parameters, corresponding wavevectors extracted from the maxima of the curves, and the resulting spin-spiral wavelengths $\lambda = {}^{2\pi}/_{q}$ are given in Table 1. For the chains with antiferromagnetic Heisenberg exchange ($J_{\mathrm{NN}} < 0$) q varies only weakly in the range of ${}^{3\pi}/_{4d_{\mathrm{NN}}} < q < {}^{\pi}/_{d_{\mathrm{NN}}}$. Nevertheless, due to the variation of the NN distance, λ can be tuned over a rather large range. Even larger wavelengths are expected for the chains with zero and positive (ferromagnetic) Heisenberg exchange. For the purely DM driven spin-spiral ($J_{\mathrm{NN}} = 0$) q is exactly ${}^{\pi}/_{2d_{\mathrm{NN}}}$ corresponding to a 90° rotation of neighboring spins. The largest wavelength is achieved for the NN distance with ferromagnetic Heisenberg exchange ($J_{\mathrm{NN}} > 0$).

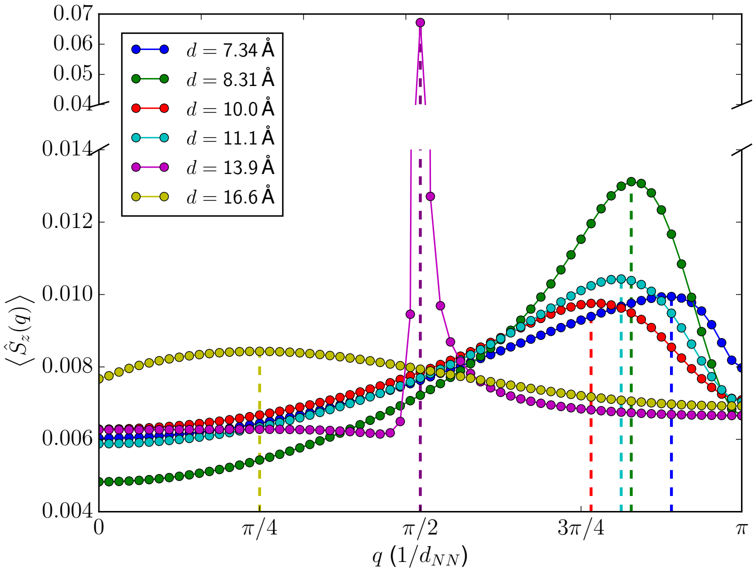

Supplementary Figure 16 | DMRG calculated spin-structure factor $\langle \hat{S}_z(q) \rangle$ for chains with different couplings. The spin-structure factors have been calculated assuming the experimentally accessible Heisenberg and DM couplings in chains with NN distances d_{NN} given in the legend. The corresponding coupling parameters and the resulting q and λ are given in Supplementary Table 1.

d_{NN} (in Å)	J_{NN} (in μeV)	$D_{\perp,\mathrm{NN}}$ (in μeV)	q (in $1/d_{\mathrm{NN}}$)	λ (in d_{NN})	λ (in Å)
7.34	-30	+20	$114/128\ \pi$	2.25	16.5
8.31	-60	-50	$106/128\ \pi$	2.41	20.1
10.0	-15	-25	$98/128\ \pi$	2.61	26.1
11.1	-25	+30	$104/128\ \pi$	2.46	27.3
13.9	0	+25	$1/2\ \pi$	4.00	55.6
16.6	+15	-20	$1/4\ \pi$	8.00	132.8

Supplementary Table 1 | Experimentally determined Heisenberg (J_{NN}) and DM ($D_{\perp,\mathrm{NN}}$) interactions extracted from the investigation of pairs of an Fe-hydrogen complex and an Fe atom (both adsorbed on the hcp site) with the distance of d_{NN}. The data is taken from Ref.1. Note that the sign of $D_{\perp,\mathrm{NN}}$ follows from the KKR calculation. The wavevectors q maximizing $\langle \hat{S}_z(q) \rangle$ and corresponding wavelengths λ result from the DMRG calculation of Supplementary Figure 16.

Supplementary References

1. Khajetoorians, A. A. *et al.* Tailoring the chiral magnetic interaction between two individual atoms. *Nature Communications* **7**, 10620 (2016). URL `http://www.nature.com/doifinder/10.1038/ncomms10620`.

2. Ternes, M. Spin excitations and correlations in scanning tunneling spectroscopy. *New Journal of Physics* **17**, 63016 (2015). URL `http://dx.doi.org/10.1088/1367-2630/17/6/063016`. `1505.04430`.

3. Meier, F., Zhou, L., Wiebe, J. & Wiesendanger, R. Revealing magnetic interactions from single-atom magnetization curves. *Science* **320**, 82–6 (2008). URL `http://www.ncbi.nlm.nih.gov/pubmed/18388289`.
